# Secretion of a foreign protein from budding yeasts is enhanced by cotranslational translocation and by suppression of vacuolar targeting

**DOI:** 10.1186/s12934-014-0125-0

**Published:** 2014-08-28

**Authors:** Ivy Fitzgerald, Benjamin S Glick

**Affiliations:** Graduate Program in Biophysical Sciences, University of Chicago, 929 East 57th Street, Chicago, Illinois 60637 USA; Department of Molecular Genetics and Cell Biology, University of Chicago, 920 East 58th Street, Chicago, Illinois 60637 USA

**Keywords:** Yeasts, Secretion, Heterologous protein production, Signal sequence, Alpha-factor, Translocation, Sorting, Vacuole, Ost1, Vps10

## Abstract

**Background:**

Budding yeasts are often used to secrete foreign proteins, but the efficiency is variable. To identify roadblocks in the yeast secretory pathway, we used a monomeric superfolder GFP (msGFP) as a visual tracer in *Saccharomyces cerevisiae* and *Pichia pastoris*.

**Results:**

One roadblock for msGFP secretion is translocation into the ER. Foreign proteins are typically fused to the bipartite α-factor secretion signal, which consists of the signal sequence followed by the pro region. The α-factor signal sequence directs posttranslational translocation. For msGFP, posttranslational translocation is inefficient with the α-factor signal sequence alone but is stimulated by the pro region. This requirement for the pro region can be bypassed by using the Ost1 signal sequence, which has been shown to direct cotranslational translocation. A hybrid secretion signal consisting of the Ost1 signal sequence followed by the α-factor pro region drives efficient translocation followed by rapid ER export. A second roadblock for msGFP secretion in *S. cerevisiae* occurs during exit from the Golgi, when some of the msGFP molecules are diverted to the vacuole. Deletion of the sorting receptor Vps10 prevents vacuolar targeting of msGFP at the expense of missorting vacuolar hydrolases such as carboxypeptidase Y (CPY) to the culture medium. However, a truncation of Vps10 blocks vacuolar targeting of msGFP while permitting CPY to be sorted normally.

**Conclusions:**

With budding yeasts, if the secretion or processing of a foreign protein is poor, we recommend two options. First, use the Ost1 signal sequence to achieve efficient entry into the secretory pathway while avoiding the processing issues associated with the α-factor pro region. Second, truncate Vps10 to suppress diversion to the vacuole. These insights obtained with msGFP highlight the value of applying cell biological methods to study yeast secretion.

**Electronic supplementary material:**

The online version of this article (doi:10.1186/s12934-014-0125-0) contains supplementary material, which is available to authorized users.

## Background

Budding yeasts such as *Saccharomyces cerevisiae* and *Pichia pastoris* are widely used as hosts to produce foreign proteins for research and therapeutic purposes [1-5]. Many of these foreign proteins traverse the secretory pathway [6,7]. Entry into the secretory pathway requires an N-terminal signal sequence that directs translocation into the endoplasmic reticulum (ER). A typical signal sequence includes a stretch of hydrophobic residues, followed by a cleavage site that is recognized by signal peptidase in the ER lumen. After entry into the ER, a protein folds, and may undergo additional changes that include disulfide bond formation, glycosylation, and oligomerization. Finally, the protein is delivered from the ER to the Golgi apparatus and then to the extracellular space. For different foreign proteins expressed in yeasts, the level of secreted product varies widely, presumably because specific steps in the secretory pathway can be inefficient [3]. Much interest is focused on identifying and overcoming these roadblocks.

One source of variability is the secretion signal. A number of signal sequences have been used to secrete foreign proteins from yeasts, but the most popular is the secretion signal from *S. cerevisiae* pre-pro-α-factor, which is the precursor to a peptide mating pheromone [1,8,9]. Pre-pro-α-factor contains a 19-residue signal sequence that terminates in a signal peptidase cleavage site. Following the signal sequence is a 66-residue pro region, which is removed in the late Golgi by the Kex2 endoprotease [10]. Finally, downstream of the dibasic Kex2 cleavage signal is an EAEA tetrapeptide, which is trimmed by the dipeptidyl aminopeptidase Ste13 [11]. Efficient secretion of foreign proteins requires the entire pre-pro-α-factor secretion signal, with or without the downstream EAEA tetrapeptide, but the secreted products are often heterogeneous due to incomplete processing by Kex2 or Ste13 [1,5].

The reason for the effectiveness of the complete pre-pro-α-factor secretion signal has been unclear. Standard assays for protein secretion simply measure the amount of product that reaches the culture medium. To examine earlier steps in the secretory pathway, we employed a monomeric superfolder variant of green fluorescent protein (msGFP) as a model foreign protein. Visualization of intracellular msGFP indicated that with the α-factor signal sequence, which mediates posttranslational translocation across the ER membrane [12,13], the translocation step is inefficient. More efficient posttranslational translocation was obtained by inclusion of the α-factor pro region. Based on this insight, we used the Ost1 signal sequence to drive cotranslational translocation of msGFP [14], thereby bypassing the pro region and its associated complications.

After entering and exiting the ER, a protein can nevertheless fail to be secreted if it is diverted to the vacuole. Budding yeasts contain a vacuolar sorting receptor called Vps10 that recognizes the carboxypeptidase Y (CPY) precursor as well as certain misfolded proteins [15-20]. When msGFP was targeted to the secretory pathway in *S. cerevisiae*, some of the msGFP molecules reached the vacuole in a Vps10-dependent manner, confirming a report that the quality control function of Vps10 extends to folded GFP [21]. Building on a previous functional dissection of Vps10 [15], we found that deletion of a single domain of Vps10 prevented diversion of msGFP to the vacuole without causing missorting of CPY. This approach may enable the engineering of yeast strains that efficiently secrete foreign proteins while preserving normal vacuolar function.

## Results

### The pro region enhances secretion even in the absence of Erv29

GFP folds poorly in the ER lumen due to intermolecular disulfide bond formation, but this problem can be overcome by using superfolder variants [22,23]. We chose a monomeric superfolder GFP (msGFP) as a reporter to track passage through the yeast secretory pathway.

**Figure 1:**
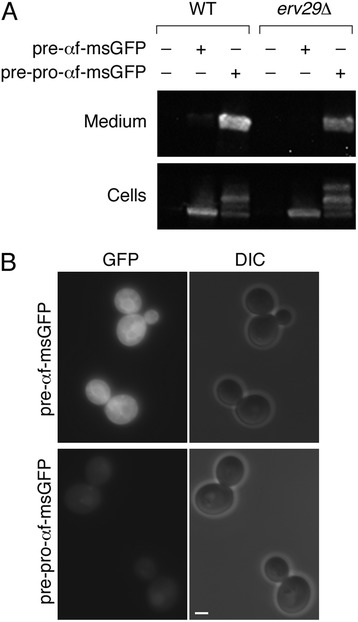
**Effects on secreted msGFP constructs of including the α-factor pro region after the α-factor signal sequence. (A)** *S. cerevisiae* cells, either expressing wild-type Erv29 (“WT”) or carrying an *erv29Δ* allele, were engineered to express msGFP fused to either the α-factor signal sequence alone (pre-αf-msGFP) or the complete α-factor secretion signal (pre-pro-αf-msGFP). Samples of the culture medium and cells were analyzed by SDS-PAGE, immunoblotting, and chemiluminescence to detect msGFP. **(B)** The strains expressing wild-type Erv29 plus the indicated msGFP constructs were imaged by fluorescence microscopy to detect GFP, and by differential interference contrast (DIC) microscopy to detect the cells. Representative cells are shown. Exposure times for the fluorescence images were 100 msec. Scale bar, 2 μm.

**Figure 2:**
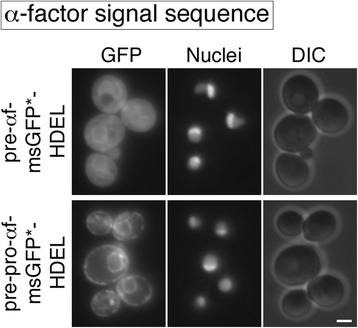
**Effects on ER-retained msGFP constructs of including the α-factor pro region after the α-factor signal sequence.** *S. cerevisiae* cells carrying *erv29Δ* and *htm1Δ* alleles and expressing nuclear-targeted DsRed-Express2 were engineered to express msGFP with a C-terminal HDEL signal, fused to either the α-factor signal sequence alone (pre-αf-msGFP*-HDEL) or the complete α-factor secretion signal (pre-pro-αf-msGFP*-HDEL). Strains were imaged by fluorescence microscopy with 300 msec exposure times to detect GFP and DsRed, and by differential interference contrast (DIC) microscopy to detect the cells. Scale bar, 2 μm.

The first goal was to determine whether the α-factor pro region enhances secretion of msGFP. For this purpose, we fused msGFP either downstream of the α-factor signal sequence alone to yield pre-αf-msGFP, or downstream of the entire α-factor secretion signal to yield pre-pro-αf-msGFP. These constructs were expressed in *S. cerevisiae* using the strong constitutive *TPI1* promoter. msGFP secretion into the medium was detected by immunoblotting. As shown in Figure 1A, almost no secretion of msGFP was seen with pre-αf-msGFP, but robust secretion was seen with pre-pro-αf-msGFP. Subsequent experiments indicated that total expression levels were not significantly affected by the presence of the pro region (see Figure 2 below). Thus, secretion of msGFP is stimulated by the α-factor pro region.

The only previously characterized function of the α-factor pro region is to interact with the ER export receptor Erv29 [24]. We therefore hypothesized that Erv29-dependent acceleration of ER export was responsible for the ability of the pro region to enhance secretion of msGFP. To test this idea, the experiment was repeated with an *erv29Δ* strain. This mutation reduced secretion of pre-pro-αf-msGFP, but surprisingly, pre-pro-αf-msGFP was still secreted much better than pre-αf-msGFP (Figure 1A). This result indicates that the enhancement of secretion by the pro region is largely independent of Erv29.

To identify the roadblock in secretion with the pre-αf-msGFP construct, we examined the cells by fluorescence microscopy. The results were striking: with pre-αf-msGFP, strong cytosolic fluorescence was seen, whereas with pre-pro-αf-msGFP, the cytosolic signal was much weaker (Figure 1B). A likely interpretation is that the pro region facilitates translocation of msGFP across the ER membrane.

### The pro region enhances translocation into the ER

As a direct test of whether pre-αf-msGFP is translocated across the ER membrane less efficiently than pre-pro-αf-msGFP, we modified the proteins to be retained in the ER by means of a C-terminal HDEL tetrapeptide [25]. Our experience is that residues preceding an HDEL signal can influence its efficacy, so to generate a signal that is known to cause ER retention [26], we also replaced the C-terminal peptide of msGFP with the wild-type GFP C-terminal peptide to create msGFP*-HDEL constructs. Removal of the signal sequence in the ER will yield either msGFP*-HDEL in the case of pre-αf-msGFP*-HDEL, or pro-αf-msGFP*-HDEL in the case of pre-pro-αf-msGFP*-HDEL. The latter construct introduces two complications. First, Erv29 will recognize the pro region and direct rapid ER export, thereby potentially compromising ER retention. To avoid this problem, we used an *erv29Δ* strain. Second, the three N-linked oligosaccharides in the pro region [27] will eventually be processed by Htm1 to trigger ER-associated degradation [28]. To avoid this problem, we introduced the *htm1Δ* mutation. Finally, to identify the ER, which includes a prominent nuclear envelope ring [29-31], we integrated a construct for expressing DsRed-Express2 fused to a nuclear localization signal [32].

The pre-αf-msGFP*-HDEL construct generated a strong cytosolic signal plus a weak ER signal (Figure 2). By contrast, the pre-pro-αf-msGFP*-HDEL construct generated a weak cytosolic signal plus a strong ER signal (Figure 2). This result confirms that inclusion of the pro region downstream of the α-factor signal sequence stimulates translocation of msGFP into the ER. We infer that posttranslational translocation of msGFP is inefficient with the α-factor signal sequence alone, and that inclusion of the pro region increases the amount of msGFP that ultimately crosses the ER membrane.

### The cotranslational Ost1 signal sequence bypasses the need for the pro region

**Figure 3:**
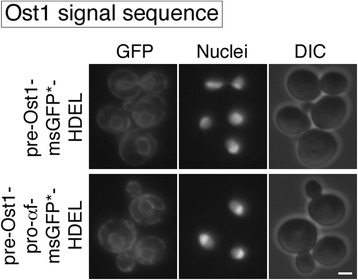
**Effects on ER-retained msGFP constructs of including the α-factor pro region after the Ost1 signal sequence.** This experiment was performed in parallel with that of Figure 2 with the same parameters, except that msGFP with a C-terminal HDEL signal was fused to either the Ost1 signal sequence alone (pre-Ost1-msGFP*-HDEL) or the Ost1 signal sequence followed by the α-factor pro region (pre-Ost1-pro-αf-msGFP*-HDEL). Scale bar, 2 μm.

For msGFP secretion, an alternative signal sequence that directs cotranslational translocation might be more effective than the α-factor signal sequence. Cleavable signal sequences in yeast typically direct posttranslational translocation [12,33], but an exception is the *S. cerevisiae* Ost1 signal sequence, which has been shown to direct cotranslational translocation [14,34]. We therefore repeated the msGFP*-HDEL experiment after replacing the α-factor signal sequence with the Ost1 signal sequence. As shown in Figure 3, both pre-Ost1-msGFP*-HDEL and pre-Ost1-pro-αf-msGFP*-HDEL generated fluorescent ER compartments with little cytosolic background. Thus, for targeting msGFP to the secretory pathway, the cotranslational Ost1 signal sequence bypasses the requirement for the α-factor pro region.

**Figure 4:**
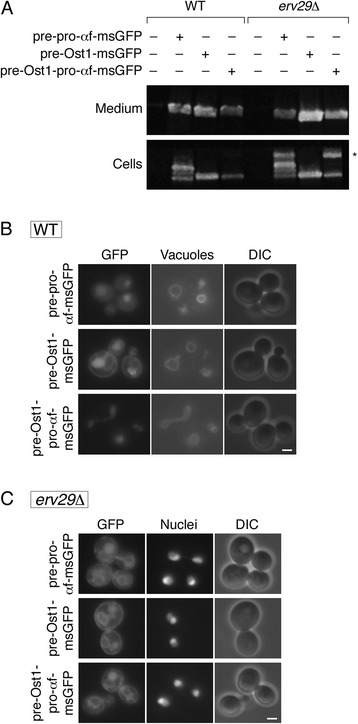
**Effects on secreted msGFP constructs of including the α-factor pro region after the Ost1 signal sequence. (A)** The analysis was performed as in Figure 1A, except that msGFP was fused to either the complete α-factor secretion signal (pre-pro-αf-msGFP), or the Ost1 signal sequence alone (pre-Ost1-msGFP), or the Ost1 signal sequence followed by the α-factor pro region (pre-Ost1-pro-αf-msGFP). The asterisk marks a band that may represent ER-localized msGFP molecules fused to the pro region. **(B)** The analysis was performed as in Figure 1B, except with the strains described in (A). Cells were stained with FM 4-64 to visualize the vacuolar membrane. Exposure times for the fluorescence images were 3oo msec. Scale bar, 2 μm. **(C)** The analysis was performed as in (B), except that vacuoles were not labeled, and the cells carried an *erv29Δ* allele and expressed nuclear-targeted DsRed-Express2. Exposure times for the fluorescence images were 300 msec. Scale bar, 2 μm.

To verify this conclusion, we examined secretion of msGFP with the Ost1 signal sequence. As shown in Figure 4A, for wild-type or *erv29Δ* cells, the amount of msGFP in the culture medium with pre-Ost1-msGFP was at least as high as the amount with pre-pro-αf-msGFP. Inclusion of the α-factor pro region after the Ost1 signal sequence to create pre-Ost1-pro-αf-msGFP did not enhance secretion, and was actually inhibitory (Figure 4A). These results indicate that for msGFP, cotranslational translocation with the Ost1 signal sequence alone is sufficient for secretion.

Additional insight came from fluorescence microscopy of strains expressing the secreted msGFP constructs. pre-Ost1-msGFP generated fluorescent nuclear envelope and cortical structures typical for the ER, as well as large solid fluorescence signals that corresponded to the vacuolar lumen (Figure 4B). We infer that cotranslational translocation of pre-Ost1-msGFP resulted in delivery to the ER lumen, and that some of the msGFP molecules were then targeted to the vacuole. The pattern was different with pre-pro-αf-msGFP and pre-Ost1-pro-αf-msGFP, which generated only vacuolar fluorescence (Figure 4B), presumably because Erv29 recognized the pro region and mediated rapid ER export. Indeed, in an *erv29Δ* strain, pre-pro-αf-msGFP and pre-Ost1-pro-αf-msGFP showed not only a vacuolar signal, but also an ER signal (Figure 4C). ER-localized msGFP molecules that retained the pro region probably account for a high-molecular weight species detected in *erv29Δ* cells but not in wild-type cells (Figure 4A, asterisk). In sum, the Ost1 signal sequence confers efficient translocation of msGFP into the ER, and inclusion of the α-factor pro region confers rapid Erv29-dependent ER exit, but rapid ER exit does not enhance secretion under our experimental conditions.

### A partial deletion of Vps10 prevents vacuolar targeting of msGFP

**Figure 5:**
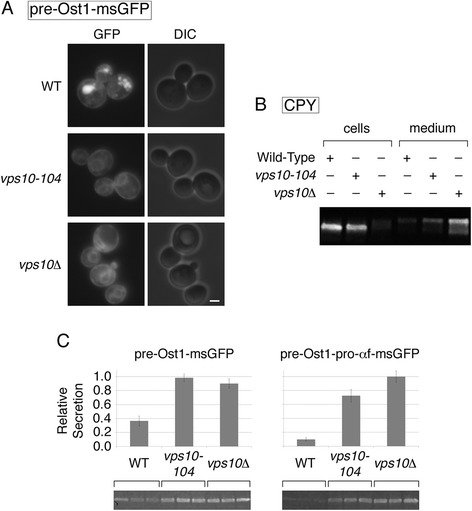
**Effects on secreted msGFP constructs of deleting or mutating Vps10. (A)** *S. cerevisiae* cells, either expressing wild-type Vps10 (“WT”) or carrying a *vps10-104* or *vps10Δ* allele, were engineered to express msGFP fused to the Ost1 signal sequence (pre-Ost1-msGFP). Exposure times for the fluorescence images were 500 msec. Scale bar, 2 μm. **(B)** Intracellular and extracellular CPY for the indicated strains was analyzed by SDS-PAGE and immunoblotting as described in Methods. **(C)** msGFP secretion was analyzed as in Figure 1A for the indicated strains, except that immunoblotting was performed with the quantitative procedure described in Methods. The experiment was done twice, with three replicates each time. Immunoblots from one of the experiments are shown. Error bars represent s.e.m.

The vacuolar accumulation of msGFP resembles previous observations that GFP variants were targeted to the yeast vacuole [21,35]. Intriguingly, vacuolar targeting of GFP was shown to depend on the sorting receptor Vps10 [21]. We therefore engineered a *vps10Δ* strain, and then repeated the fluorescence microscopy analysis with pre-Ost1-msGFP (Figure 5A). The vacuolar signal was virtually abolished by the *vps10Δ* mutation, leaving only an ER signal. This result confirms that Vps10 can recognize folded GFP for delivery to the vacuole.

The N-terminal lumenal portion of Vps10 consists of two related domains termed domains 1 and 2, and selective removal of domain 1 to create the *vps10-104* mutation reportedly preserved the localization, stability, and vacuolar hydrolase sorting capacity of Vps10 [15]. No ligands for domain 1 have been described. We speculated that domain 1 might recognize protein structures that are not normally present in the yeast secretory pathway, in which case removal of domain 1 with the *vps10-104* mutation might prevent targeting of msGFP to the vacuole while permitting correct sorting of vacuolar hydrolases. Indeed, in a *vps10-104* strain, pre-Ost1-msGFP generated an ER signal but no vacuolar signal, a pattern indistinguishable from that seen in the *vps10Δ* strain (Figure 5A). Immunoblotting of intracellular and secreted carboxypeptidase Y (CPY), which is a ligand for Vps10, confirmed that CPY was missorted to the culture medium in the *vps10Δ* strain but was largely retained intracellularly in the *vps10-104* strain (Figure 5B) [17,20]. Thus, a selective mutation in Vps10 can suppress targeting of a foreign protein to the vacuole while preserving other aspects of vacuolar function.

Elimination of vacuolar targeting would be expected to increase the secretion of msGFP. We compared the secretion of msGFP from wild-type, *vps10Δ*, and *vps10-104* strains expressing either pre-Ost1-msGFP or pre-Ost1-pro-αf-msGFP (Figure 5C). The *vps10Δ* and *vps10-104* mutations boosted secretion ~2.5-fold for pre-Ost1-msGFP and ~8-fold for pre-Ost1-pro-αf-msGFP. These results confirm that suppression of vacuolar targeting increased the secretion efficiency of a model protein.

### Translocation into the ER can be a roadblock in secretion with *Pichia pastoris*

**Figure 6:**
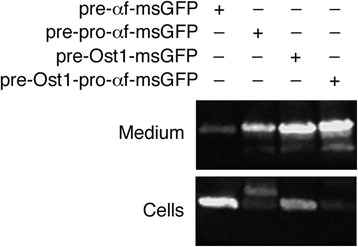
**Effects of the different signal sequences on secretion of msGFP from** ***P. pastoris*** **.** Strains of *P. pastoris* were engineered to express msGFP fused to either the α factor signal sequence alone (pre-αf-msGFP), or the complete α factor secretion signal (pre-pro-αf-msGFP), or the Ost1 signal sequence alone (pre-Ost1-msGFP), or the Ost1 signal sequence followed by the α factor pro region (pre-Ost1-pro-αf-msGFP). Extracellular and intracellular msGFP was analyzed as in Figure 1A.

**Figure 7:**
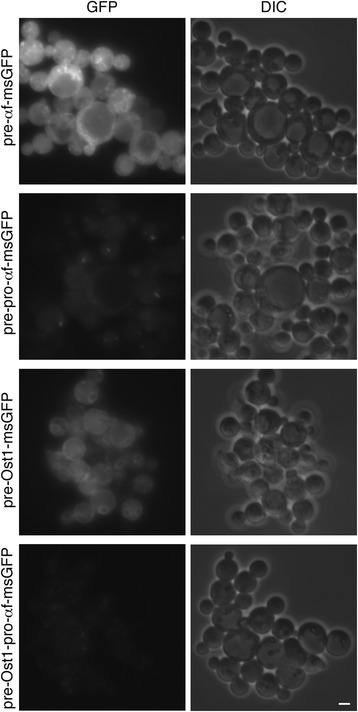
**Effects of the different signal sequences on intracellular accumulation of msGFP in** ***P. pastoris*** **.** Strains of *P. pastoris* expressing the indicated msGFP constructs (see Figure 6) were imaged by fluorescence microscopy to detect GFP, and by differential interference contrast (DIC) microscopy to detect the cells. Representative groups of cells are shown. Exposure times for the fluorescence images were 200 msec. Scale bar, 2 μm.

As an indication of whether this analysis can be generalized to other budding yeasts, we expressed different msGFP constructs in *P. pastoris*. Cells were transformed with integrating vectors that used the methanol-inducible *AOX1* promoter [36,37] to express pre-αf-msGFP, or pre-pro-αf-msGFP, or pre-Ost1-msGFP, or pre-Ost1-pro-αf-msGFP. Secretion of msGFP was weak with the α-factor signal sequence alone, and was stronger with the complete α-factor secretion signal (Figure 6). Even stronger secretion was seen with the Ost1 signal sequence, either alone or followed by the α-factor pro region (Figure 6). The hybrid secretion signal consisting of the Ost1 signal sequence plus the α-factor pro region yielded robust secretion of msGFP with minimal accumulation in the cells. Fluorescence microscopy revealed that expression of pre-αf-msGFP generated mainly cytosolic fluorescence, whereas expression of pre-Ost1-msGFP generated ER fluorescence (Figure 7). Very little cellular fluorescence was seen with the pre-pro-αf-msGFP construct, and even less with the pre-Ost1-pro-αf-msGFP contruct (Figure 7). *P. pastoris* showed negligible vacuolar fluorescence with the msGFP constructs, perhaps because *P. pastoris* Vps10 has a low affinity for msGFP, but otherwise these results are similar to those seen with *S. cerevisiae*.

The data obtained with *P. pastoris* support three conclusions about msGFP: (1) Cotranslational translocation driven by the Ost1 signal sequence was more efficient than posttranslational translocation driven by the α-factor signal sequence. (2) The α-factor pro region stimulated posttranslational translocation when fused downstream of the α-factor signal sequence. (3) The α-factor pro region stimulated ER export when fused downstream of the signal sequence from either α-factor or Ost1.

## Discussion

One motivation for studying the yeast secretory pathway is to engineer this system for improved secretion of foreign proteins. Here, we used msGFP as a model secretory protein, reasoning that fluorescence would enable us to visualize roadblocks that are hard to detect by other means. Our analysis employed standard tools of cell biological analysis. With luck, approaches that led to better msGFP secretion under these laboratory conditions will be applicable to other foreign proteins under industrial conditions.

We began by testing the α-factor secretion signal, consisting of the signal sequence plus pro region, because wild-type and mutant versions of this signal are commonly used to secrete foreign proteins [1,2,38,39]. Inclusion of the α-factor pro region was reportedly needed for high-level secretion of human insulin-like growth factor 1 (IGF1) from *S. cerevisiae* [40] and of human lysozyme from *P. pastoris* [9]. Our experiments with msGFP fusion proteins expressed in *S. cerevisiae* and *P. pastoris* revealed that the α-factor signal sequence alone yielded minimal secretion, while the full-length α-factor secretion signal yielded efficient secretion. Thus, the α-factor pro region has repeatedly been found to stimulate secretion, but the mechanistic basis for this effect has been unclear.

One possibility is that the pro region enhances secretion by accelerating export from the ER. Many proteins, including pre-pro-α-factor, leave the ER by receptor-dependent pathways [41,42]. ER export of pre-pro-α-factor involves recognition of the pro region by the transmembrane receptor Erv29 [24,41]. However, we found that with an *erv29Δ* strain of *S. cerevisiae*, the full-length α-factor secretion signal was still much more effective than the α-factor secretion signal alone at driving secretion. This result pointed to a different explanation for the ability of the pro region to enhance secretion of msGFP.

Our attention then turned to translocation, because pre-pro-α-factor crosses the ER membrane in a posttranslational manner, and fusion proteins with the α-factor signal sequence presumably follow the same pathway [12,13,43]. Posttranslational translocation into the ER is thought to employ a Brownian ratchet, in which the polypeptide chain slides back and forth in the translocon and is captured in the lumen by binding of the Hsp70 chaperone Kar2 (BiP) [44]. Posttranslational translocation can be slowed or blocked by the presence of a folded domain in the cytosol [45]. Based on the folding pathway of GFP [46], we speculate that initial folding of immature msGFP impedes translocation, and that more stable folding upon chromophore maturation blocks translocation entirely.

According to this hypothesis, the α-factor pro region enhances secretion of msGFP by stimulating translocation into the ER. Our fluorescence microscopy data favor this idea. When msGFP was fused to the α-factor signal sequence alone, fluorescent msGFP accumulated in the cytosol, but when msGFP was fused to the full-length α-factor secretion signal, much less cytosolic fluorescence was seen. To focus specifically on the ER translocation step, we appended an HDEL retention signal to msGFP. As expected, fusion of the α-factor signal sequence alone to HDEL-tagged msGFP generated mainly cytosolic fluorescence, whereas fusion of the full-length α-factor secretion signal to HDEL-tagged msGFP generated ER fluorescence. These findings are reminiscent of an earlier report that with *S. cerevisiae*, fusion of the α-factor signal sequence or two other signal sequences to IGF1 resulted in intracellular accumulation, whereas fusion of the full-length α-factor secretion signal to IGF1 resulted in secretion [40]. Therefore, inclusion of the α-factor pro region apparently enables msGFP and other foreign proteins to enter the yeast ER.

How does the pro region stimulate translocation? The answer is unknown, but the pro region may simply provide a long stretch of unfolded polypeptide upstream of the folded passenger protein, thereby allowing Kar2 to act as an effective ratchet. The three N-linked oligosaccharides that are added to the pro region [27] may further promote directional translocation by hindering back-sliding of the polypeptide chain in the translocon.

This analysis prompted us to test a cotranslational translocation pathway, which would permit msGFP to cross the ER membrane prior to folding [45]. Cotranslational translocation into the yeast ER occurs when the signal sequence is sufficiently hydrophobic [12,13]. Only a small fraction of cleavable yeast signal sequences meet this criterion [33], but the hydrophobic Ost1 signal sequence has been rigorously shown to direct cotranslational translocation [14,34]. When fused to msGFP, the Ost1 signal sequence yielded efficient translocation as well as efficient secretion. Inclusion of the α-factor pro region after the Ost1 signal sequence did not stimulate translocation. These results confirm that with msGFP, the requirement for the pro region can be bypassed by using a signal sequence that directs cotranslational translocation.

We propose that the Ost1 signal sequence, or similar hydrophobic signal sequences, may prove generally useful for secreting foreign proteins. Compared to the α-factor secretion signal, the Ost1 signal sequence has two advantages. First, because translocation with the Ost1 signal sequence is cotranslational, even rapidly folding proteins should enter the ER efficiently. By contrast, with the α-factor secretion signal, some rapidly folding proteins might be trapped in the cytosol despite the presence of the pro region. Second, because the Ost1 signal sequence requires cleavage only by signal peptidase, the N-termini of the mature proteins are likely to be homogeneous. By contrast, with the α-factor secretion signal, proteolytic removal of the pro region or the downstream EAEA peptide is often incomplete. On the other hand, a potential concern with the Ost1 signal sequence is that when fusion proteins are expressed at very high levels, the cotranslational targeting pathway might be overwhelmed. Empirical tests will be needed to assess the practical value of the Ost1 signal sequence.

A foreign protein that crosses the ER membrane must then be exported to the Golgi. As described above, ER export can be accelerated by receptors such as Erv29, which recognizes the α-factor pro region. In both *S. cerevisiae* and *P. pastoris*, we saw accumulation of msGFP in the ER with the Ost1 signal sequence alone, but not with the Ost1 signal sequence followed by the α-factor pro region, suggesting that the pro region accelerated ER export. This effect of the pro region was abolished in *S. cerevisiae* by deleting Erv29. Yet inclusion of the pro region after the Ost1 signal sequence did not enhance secretion of msGFP, presumably because receptor-independent “bulk flow” export from the ER is quite efficient [47]. It is possible that at very high expression levels, Erv29-dependent ER export could aid secretion by preventing excess protein accumulation in the ER, in which case a hybrid secretion signal consisting of the Ost1 signal sequence followed by the α-factor pro region may prove to be useful.

Another potential roadblock in secretion occurs during export from the Golgi. Yeast cells have a quality control system in which the vacuolar sorting receptor Vps10 targets misfolded proteins from the Golgi to the vacuole [15,18,19]. Vps10 can also recognize folded proteins such as GFP and α-1 antitrypsin [21,48], suggesting that Vps10 acts broadly to capture proteins that display conformations not normally found in the secretory pathway. The implication is that for some foreign proteins, deleting Vps10 might enhance secretion by preventing vacuolar targeting. Indeed, strains lacking a Vps10 homolog showed improved secretion in *P. pastoris* [49], and similar results were seen with the fission yeast *Schizosaccharomyces pombe* and the filamentous fungus *Aspergillus oryzae* [50,51]. In our studies of *S. cerevisiae*, msGFP accumulated in the vacuole, and this vacuolar accumulation was prevented by deleting Vps10.

A problem with deleting Vps10 is that CPY and some other vacuolar hydrolases will be missorted to the culture medium [15-17,20]. These secreted vacuolar hydrolases will contaminate a secreted foreign protein and may contribute to its degradation. Ideally, Vps10 would be mutated so that it continues to sort vacuolar hydrolases while no longer targeting foreign proteins to the vacuole.

**Figure 8:**
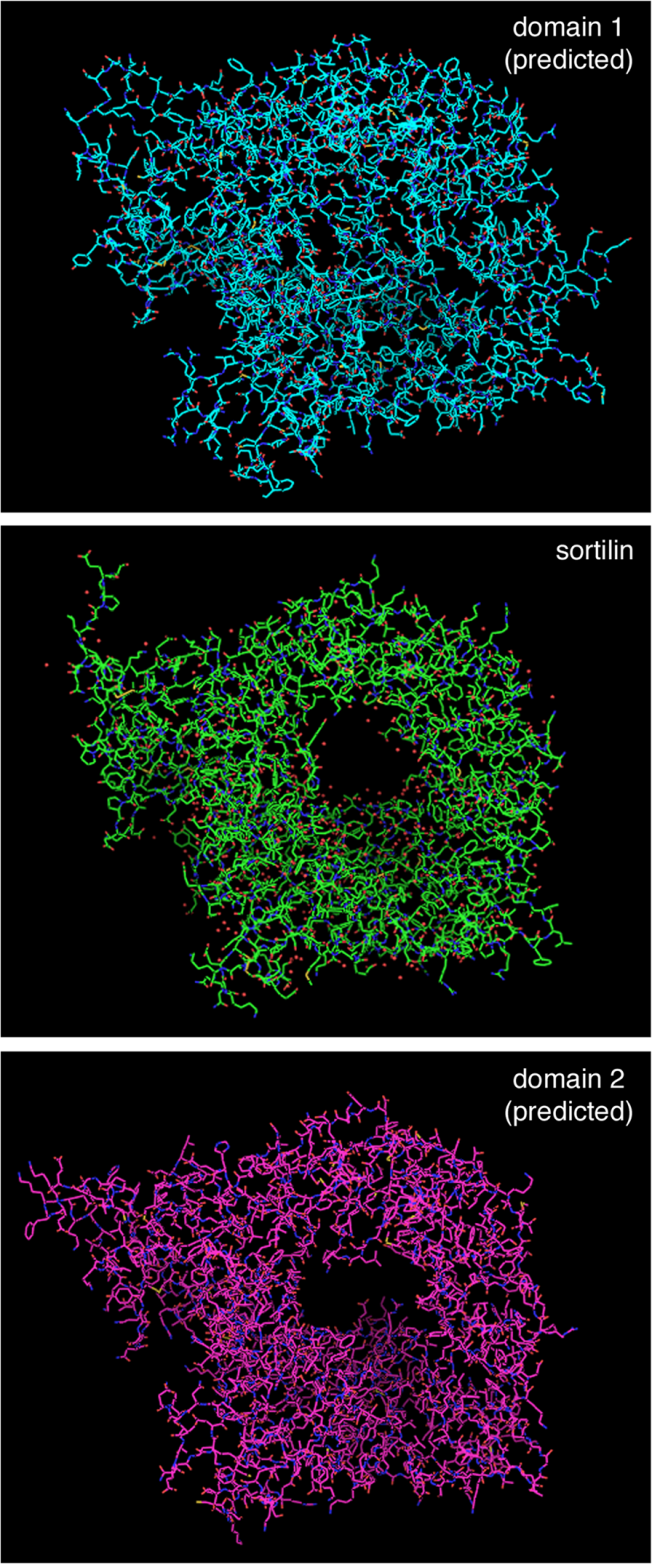
**Predicted structures of domains 1 and 2 of Vps10 compared to the known structure of sortilin.** The protein sequences of *S. cerevisiae* Vps10 domain 1 (residues 22–737) and domain 2 (residues 719–1393) were submitted to the protein homology/analogy recognition engine Phyre (http://www.sbg.bio.ic.ac.uk/phyre/html/) [54], which detected the similarity to sortilin and generated PDB files for the predicted tertiary structures. A file for the experimentally determined structure of the human sortilin lumenal domain (PDB ID 3F6K) [53] was downloaded from the National Center for Biotechnology Information. To generate the images shown, these PDB files were opened with MacPyMOL using the default settings.

To engineer such a mutation in Vps10, we built on earlier structure-function studies of the Vps10 family, which includes mammalian sortilin [52]. Fungal members of this family contain two lumenal domains termed domains 1 and 2, each of which is related to the single domain in sortilin [15]. The sortilin domain consists mainly of a ten-bladed β-propeller with a central tunnel [53]. Sequence alignment revealed that sortilin is more similar to domain 2 of *S. cerevisiae* Vps10 than to domain 1 [15]. In support of this conclusion, structure predictions obtained with the Phyre server [54] indicate that domain 2 of Vps10 is likely to resemble the sortilin domain, whereas domain 1 may have a somewhat modified fold that obscures the central tunnel (Figure 8). Previous studies of *S. cerevisiae* demonstrated that vacuolar hydrolases are sorted by domain 2, and that domain 1 could be removed with no significant effect on vacuolar function [15]. Based on these findings, we speculated that domain 2 of yeast Vps10 has a conserved role in sorting vacuolar hydrolases, while domain 1 might be a fungal-specific adaptation for recognizing abnormal protein structures.

To test this hypothesis, we replaced *S. cerevisiae* Vps10 with a truncated version lacking domain 1 [15]. The strain with truncated Vps10 sorted CPY almost normally, but like the *vps10Δ* strain, it did not accumulate msGFP in the vacuole. This result establishes msGFP as the first known ligand for domain 1 of Vps10. Truncation of Vps10 enhanced the secretion of msGFP from *S. cerevisiae*, consistent with a higher percentage of the msGFP molecules trafficking from the Golgi to the cell surface. It remains to be determined whether other folded foreign proteins are also recognized by domain 1. If so, then truncation of Vps10 is an attractive option for boosting the secretion of foreign proteins from budding yeasts, and potentially from other fungi, without substantially altering vacuolar function.

Our experiments show that cell biological studies with a model protein can suggest avenues for increasing the secretion of foreign proteins from yeasts. If a protein is poorly secreted or incompletely processed with existing approaches, we offer two recommendations. First, the Ost1 signal sequence drives efficient translocation into the ER, and avoids the incomplete processing caused by the α-factor pro region. If incomplete processing is not a concern, a hybrid secretion signal consisting of the Ost1 signal sequence followed by the α-factor pro region might be beneficial for ensuring both efficient translocation and efficient ER export. Second, selective removal of domain 1 of Vps10 can prevent a foreign protein from being diverted to the vacuole. It will be interesting to test these approaches with commercially and therapeutically relevant foreign proteins.

## Methods

### Strains and plasmids

*S. cerevisiae* strains were derivatives of the haploid strain JK9-3da (*leu2-3,112 ura3-52 rme1 trp1 his4*) [55]. These cells were grown in rich glucose medium (YPD) supplemented with 20 μg/mL each of adenine and uracil, or in minimal glucose dropout medium (SD) [56] prepared using nutrient mixtures from Sunrise Science Products. *P. pastoris* strains were derivatives of the haploid strain PPY12 (*his4 arg4*) [57]. These cells were grown as precultures in YPD, then transferred to minimal glycerol medium containing 0.05% yeast extract (SYG), then transferred to minimal methanol medium containing 0.05% yeast extract (SYM) [37]. All yeast cultures were grown with shaking at 200 rpm in baffled flasks.

Molecular biology procedures were simulated and recorded using SnapGene software (GSL Biotech), and the supplementary information includes a folder of annotated plasmid sequence/map files that can be opened with SnapGene Viewer (http://www.snapgene.com/products/snapgene_viewer/) [see Additional file 1]. msGFP was derived from enhanced GFP by introducing superfolder mutations [23], as well as the monomerizing A206K mutation [58] plus modified N- and C-terminal peptides. A full characterization of msGFP will be provided elsewhere (manuscript in preparation), but the sequence of msGFP is available in the supplementary SnapGene files. For constitutive expression in *S. cerevisiae*, msGFP was fused to the α-factor or Ost1 signal sequence, with or without the α-factor pro region. For retention in the ER, the C-terminal peptide of msGFP was replaced with the wild-type GFP C-terminal peptide plus HDEL [26]. Constructs were expressed using the *TPI1* promoter and *CYC1* terminator in the *TRP1* integrating vector YIplac204 [59]. For regulated expression in *P. pastoris*, msGFP constructs were transferred into the *HIS4* integrating vector pIB4, which contains the methanol-inducible *AOX1* promoter [37]. Integrating vectors were linearized with restriction enzymes as indicated in the supplementary SnapGene files, and were then transformed into *S. cerevisiae* using lithium acetate [60] or into *P. pastoris* using electroporation [37]. Transformants were selected on minimal dropout plates, with histidine, leucine, or tryptophan omitted as needed to select for integration of the linearized vectors. To avoid multi-copy integrants, a number of clones from each transformation were screened by fluorescence microscopy, and clones with unusually high fluorescence signals were excluded from further analysis.

Gene deletions were performed using standard methods. Briefly, to delete the *S. cerevisiae ERV29* gene, the *kanMX* cassette was amplified from pFA6a-kanMX6 [61] using primers GACTCAAAAAAAGTGAAAACAAAACTGAAAGGATAGATCACGTACGCTGCAGGTCGAC and GAGTGAACAGAAGGGACATAAAGAAAAGATTTCCTTTACAATATCGATGAATTCGAGCTCG. The resulting fragment was transformed into cells, which were plated on YPD medium containing 250 μg/ml G418 (Sigma-Aldrich) to select for double-crossover replacement of the *ERV29* open reading frame. A similar approach was used to delete the *S. cerevisiae HTM1* gene, except that a hygromycin resistance cassette was amplified from pAG32 [62] using primers GAGTAACCATGATAATTTCATATTTCCATGGATTGGTTACATTCAGGGAAATAGACCAGATCTGTTTAGCTTGCCTTGTCC and CATTTATTACTGGTGCCATTATGTAAAAGCTGTAGAGGTCTATCTAAAAGAGTGATTCGTTTTCGACACTGGATGGCGGCGTTAG, and selection was performed on YPD plates containing 200 μg/mL hygromycin. Each gene deletion was confirmed by two separate diagnostic PCR amplifications of genomic DNA.

### Assays for secretion of msGFP and CPY

For *S. cerevisiae*, secretion was measured in YPD because the recovery of msGFP for immunoblotting was more reliable in rich than in minimal medium. A 5-mL culture in YPD was inoculated from a preculture, and grown overnight at 30°C with shaking in a baffled flask to an OD_600_ of 0.7-0.8. Then 1.75 OD_600_ units were transferred to a 15-mL tube, washed twice with deionized water by centrifugation and resuspension, and resuspended in 5 mL of fresh YPD to an OD_600_ of 0.35. This culture was incubated with shaking at 30°C for 3 h, an incubation period that enabled secretion of a detectable amount of msGFP or CPY while the cells remained in mid-log phase. Then 1.6 mL of culture was transferred to a microcentrifuge tube. When multiple cultures were processed in parallel, 1.6 mL of the culture with the lowest OD_600_ was collected, and an equivalent number of OD_600_ units was collected from each of the other cultures. These volume adjustments were minor because all of the strains grew at similar rates. Each culture was centrifuged at 3000×g (5600 rpm) for 5 min in a microcentrifuge to separate the cells from the secreted proteins.

For *P. pastoris*, cultures were grown in YPD to an OD_600_ of ~ 0.3, then washed with SYG using a bottle-top filter, then resuspended in SYG and grown overnight. The cultures were then washed and transferred to SYM supplemented with 0.25 mg/mL bovine serum albumin at an OD_600_ of ~1.2. After 3 h in this inducing medium, the cultures were washed and transferred to fresh SYM supplemented with 0.25 mg/mL bovine serum albumin. After an additional 3 h, the secreted and intracellular proteins were separated as for *S. cerevisiae*.

To analyze msGFP or CPY in the culture medium [63], 1.5 mL of supernatant from the final centrifugation was transferred to a fresh tube, and was supplemented with 167 μL of 100% (w/v) trichloroacetic acid (TCA). This mixture was left on ice for at least 20 min. The tube was then centrifuged at 16,000 × g (maximum speed) in a microcentrifuge for 15 min at 4°C. The supernatant was carefully removed and discarded. Then the precipitated proteins were resuspended in 50 μL SDS-PAGE sample buffer supplemented with 50 mM Na^+^-PIPES, pH 7.5, and 2% β-mercaptoethanol. The samples were boiled for 5 min, then centrifuged for 2 min at 16,000 × g in a microcentrifuge to remove insoluble material. Twenty μL of the final supernatant was loaded on a gel for SDS-PAGE.

To analyze msGFP or CPY in the cells [63], the pellet from the final centrifugation was resuspended in 1 mL of deionized water and then centrifuged at 3000 × g, and the water was removed. The pellet was resuspended in 200 μL of 5% (w/v) TCA. This mixture was supplemented with 100 μL of 0.5 mm diameter glass beads (BioSpec Products), and was vortexed for 30 sec to break the cells. Eight hundred μL of 5% TCA was added and mixed. Then 800 μL of this solution was transferred to a fresh tube and left on ice for at least 5 min. The sample was centrifuged and processed as described above, except that the precipitated cellular proteins were dissolved in 400 μL SDS-PAGE sample buffer.

For immunoblotting, 20 μL of each sample was run on a 4-20% tricine gel (Bio-Rad). The separated proteins were transferred to an Immobilon membrane (Millipore). Subsequent manipulations were performed at room temperature. The membrane was blocked with 5% nonfat dry milk in TBST, which was Tris-buffered saline (TBS; 50 mM Tris–HCl, pH 7.6, 150 mM NaCl) plus 0.05% Tween 20, and was then incubated with shaking for 1 h with either 1:1000 goat polyclonal anti-GFP antibody (Applied Biological Materials) or 0.25 μg/mL mouse monoclonal anti-CPY antibody (Invitrogen; clone 10A5B5) in 1% milk/TBST. After a rinse with 5% milk/TBST, the membrane was incubated with shaking for 1 h with a 1:20,000 peroxidase-conjugated secondary antibody in 0.5% milk/TBST, either rabbit anti-goat IgG (Sigma-Aldrich) for detecting GFP or sheep anti-mouse IgG (GE Healthcare) for detecting CPY. After final rinses with 5% milk/TBST followed by TBS, the antibody-bound proteins were detected by chemiluminescence using SuperSignal West Femto Maximum Sensitivity Substrate (Thermo Scientific). Alternatively, the procedure was modified as follows for quantitative analysis of msGFP secretion. The membrane was blocked with Odyssey Blocking Buffer (LI-COR) plus 0.2% Tween 20, and the secondary antibody was IRDye 680RD donkey anti-goat (LI-COR) diluted 1:15,000 in Odyssey Blocking Buffer + 0.2% Tween 20 + 0.01% SDS. The signal was detected using a LI-COR Odyssey CLx infrared imaging system. Lanes were specified, and bands were either automatically picked or drawn by the user. The median background was subtracted from each band using pixels above and below the band area, and the integrated intensities of the bands were averaged and normalized.

### Fluorescence microscopy

For imaging intracellular msGFP, DsRed-Express2, and FM 4-64, 1.5 μL from a culture at an OD_600_ of 0.4-0.5 was compressed on a slide beneath a #1.5 coverslip. The yeast cells were viewed immediately with a Zeiss Axioplan 2 microscope using a 1.4-NA Plan-Apo objective and a FITC or Texas Red filter set (Chroma). Single-plane images were captured with a Hamamatsu digital camera using Zeiss AxioVision software. Images were resized and cropped with Adobe Photoshop, but were not otherwise processed.

Labeling of vacuoles with FM 4-64 was performed as previously described [64]. Briefly, cells were incubated with 0.8 μM FM 4-64 for 5 min, and the dye was quenched by adding 2 μM 4-sulfonato calix[8]arene, sodium salt (Biotium). The internalized dye was then chased for approximately 1 h to label the vacuolar membrane.

## Additional file

Additional file 1:
**Compressed folder of plasmid sequence/map files.** This ZIP archive contains 13 files corresponding to the plasmid constructs used in the study. The annotated sequences and maps are encoded in SnapGene's .dna format. These files can be opened with the free SnapGene Viewer (http://www.snapgene.com/products/snapgene_viewer/).

## Abbreviations

CPY: Carboxypeptidase Y
DIC: Differential interference contrast
ER: Endoplasmic reticulum
GFP: Green fluorescent protein
IGF1: Insulin-like growth factor 1
msGFP: Monomeric superfolder GFP
SYG: Minimal glycerol medium
SYM: Minimal methanol medium
TBS: Tris-buffered saline
TBST: Tris-buffered saline plus Tween 20
TCA: Trichloroacetic acid
YPD: Rich glucose medium
